# Electronegative LDL from Rabbits Fed with Atherogenic Diet Is Highly Proinflammatory

**DOI:** 10.1155/2019/6163130

**Published:** 2019-08-22

**Authors:** Po-Yuan Chang, Jou-Hsiang Pai, Yu-Sheng Lai, Shao-Chun Lu

**Affiliations:** ^1^Cardiovascular Center and Division of Cardiology, Department of Internal Medicine, National Taiwan University College of Medicine, Taipei, Taiwan; ^2^Department of Biochemistry and Molecular Biology, National Taiwan University College of Medicine, Taipei, Taiwan

## Abstract

Electronegative low-density lipoprotein (LDL(-)) has been found in the plasma of familial hypercholesterolemia and acute myocardial infarction and has been implicated in atherosclerosis and cardiovascular disease. However, less is known about the involvement of LDL(-) in atherosclerosis-related inflammation. This study aims at investigating the inducibility of LDL(-) by atherogenic diet in rabbits and at exploring the proinflammatory potential of the diet-induced LDL(-) in macrophages. Rabbits were fed with an atherogenic diet; LDL was isolated from plasma by NaBr density gradient ultracentrifugation and was then resolved into nLDL and LDL(-) by anion-exchange chromatography. Isolated nLDL and LDL(-) were directly used or incubated with 10 *μ*M CuSO_4_ for 24 h to produce copper- (Cu-) ox-nLDL and Cu-ox-LDL(-). The effects of these LDLs on inflammation were evaluated in THP-1-derived macrophages. Macrophages were treated with nLDL, LDL(-), and extensively oxidized LDL (ox-LDL), then the levels of interleukin- (IL-) 1*β*, IL-6, and tumor necrosis factor- (TNF-) *α* in a culture medium were determined by ELISA, and the levels of total and phosphorylated I*κ*B, p65, p38, JNK, and ERK in cell lysates were determined by Western blotting. The LDL(-) induced significantly higher levels of IL-1*β*, IL-6, and TNF-*α* in the medium. The levels of phosphorylated/total I*κ*B, p65, p38, JNK, and ERK were also upregulated by LDL(-). In contrast, nLDL, Cu-ox-nLDL, and Cu-ox-LDL(-) exhibited much less effect. Knockdown of lectin-type oxidized LDL receptor- (LOX-) 1 resulted in significant reduction in LDL(-)-induced IL-1*β*, IL-6, and TNF-*α*. In addition, these LDL(-) effects were also markedly attenuated by inhibition of NF-*κ*B and ERK1/2. The data suggested that LDL(-) induced inflammation through LOX-1-, NF-*κ*B-, and ERK1/2-dependent pathways. Taken together, our results show that rabbits fed with atherogenic diet produce a highly proinflammatory LDL(-) that is more potent in inducing inflammation than nLDL and extensively oxidize LDL in macrophages. The results thus provide a novel link between diet-induced hypercholesterolemia and inflammation.

## 1. Introduction

It is well known that oxidized low-density lipoprotein (LDL; ox-LDL) contributes to the pathogenesis of atherosclerosis. ox-LDL is recognized by macrophage scavenger receptors and then taken up through receptor-mediated endocytosis, ultimately leading to the formation of lipid-laden foam cells. This process is a critical event in atherosclerosis [1]. In addition to its role in lipid loading on macrophages, ox-LDL also has other biological functions, such as causing endothelial dysfunction and inducing smooth muscle cell proliferation, that contribute to the pathogenesis of atherosclerosis [2]. Accumulating evidence suggests that atherosclerosis is also a chronic inflammatory disease [3, 4]. However, the extent of involvement of ox-LDL in vascular inflammation is less understood.

Although the precise mechanism of LDL oxidation *in vivo* is not yet fully established, ox-LDL generated by exposure of LDL to Cu_2_SO_4_*in vitro* has been widely used in related studies. Only a few studies have used ox-LDL from natural sources. This could be due to the level of ox-LDL in natural sources being low and difficult to obtain, while copper- (Cu-) ox-LDL is relatively easy to prepare and is able to induce foam cell formation. In a previous study, we reported that electronegative LDL (LDL(-)) from the plasma of ST-elevated myocardial infarction (STEMI) patients induced production of interleukin- (IL-) 1*β* via the lectin-type oxidized LDL receptor- (LOX-) 1 in macrophages [5]. IL-1*β* is a central mediator of inflammation that was implicated in the development of atherosclerosis and acute myocardial infarction [6, 7]. Moreover, LDL(-), isolated from healthy normolipemic subjects, has been shown to induce production of GRO*β*, GRO*γ*, IL-6, IL-8, and MCP1 in human monocytes and lymphocytes [8–10]; induce IL-1*β* release in human monocytes and macrophages [11]; and induce production of MMP-9 and TIMP-1 in human monocytes [12]. Moreover, circulating ox-LDL was also associated with the plasma levels of tumor necrosis factor-*α* (TNF-*α*) and C-reactive protein (CRP) [13]. Those results suggested that LDL(-) has inflammatory properties. Naturally occurring LDL(-) was found to be elevated in the plasma of patients with hypercholesterolemia, type II diabetes, and STEMI [14–17], and all of those patients were associated with chronic inflammation [18–20]. Thus, LDL(-) may contribute to inflammation in these patients. In addition, our previous study showed that STEMI LDL(-) is more potent than Cu-ox-LDL in inducing IL-1*β* production by macrophages [21]. Those results suggested that the characteristics of LDL(-) and Cu-ox-LDL differed in terms of inducing inflammation.

Only a few papers have reported that plasma LDL(-) can be induced by an atherogenic diet [22, 23]; moreover, it is not clear if diet-induced LDL(-) is able to induce an inflammatory response in macrophages. Feeding cholesterol to rabbits is a widely used model for experimental atherosclerosis studies [24, 25]. We reported that an atherogenic diet induced inflammation in aortic atherosclerotic plaque and elevation of plasma ox-LDL, detected using an ox-LDL enzyme-linked immunosorbent assay (ELISA) kit, in rabbits [26]. In the present study, we isolated native LDL (nLDL) and LDL(-) from the plasma of rabbits fed an atherogenic diet. LDL(-) and nLDL were directly used or exposed to Cu_2_SO_4_*in vitro* for 24 h to produce extensively oxidized Cu-ox-nLDL and Cu-ox-LDL(-). Then, the effects of nLDL, LDL(-), Cu-ox-nLDL, and Cu-ox-LDL(-) on the production of the proinflammatory cytokines, IL-1*β*, IL-6, and tumor necrosis factor- (TNF-) *α*, and activation of nuclear factor- (NF-) *κ*B and mitogen-activated protein kinases (MAPKs) in macrophages were investigated.

## 2. Materials and Methods

### 2.1. Materials

RPMI 1640, penicillin/streptomycin, fetal bovine serum (FBS), and L-glutamine were obtained from Gibco BRL/Life Technologies (Rockville, MD, USA). Dimethyl sulfoxide (DMSO), phorbol 12-myristate 13-acetate (PMA), and 3-(4,5-dimethylthiazol-2-yl)-2,5-diphenyl tetrazolium bromide (MTT) were obtained from Sigma-Aldrich (St. Louis, MO, USA). The Beckman Paragon System was from Beckman (Palo Alto, CA, USA). Human IL-1*β*, IL-6, and TNF-*α* ELISA kits were obtained from R&D Systems (Minneapolis, MN, USA). The Beckman Paragon System was from Beckman (Palo Alto, CA). A mouse monoclonal antibody against human *β*-actin was obtained from Chemicon (Temecula, CA, USA). Antibodies against ERK1/2, inhibitor of NF-*κ*B (I*κ*B), c-Jun N-terminal kinase (JNK), p38, and p65 were purchased Santa Cruz Biotechnology (Santa Cruz, CA, USA). Antibodies against phosphorylated- (phospho-) I*κ*B were obtained from Abcam (Cambridge, UK). Antibodies against phospho-JNK, phospho-p65, phospho-extracellular signal-regulated kinase 1/2 (ERK1/2) (Thr202/204), and phospho-p38 were obtained from Cell Signaling Technology (Danvers, MA, USA). U0126 (a MEK inhibitor), SB203580 (a p38 MAPK inhibitor), SP600125 (a c-Jun N-terminal kinase inhibitor), and LY294002 (a phosphoinositide 3-kinases inhibitor) were purchased from Calbiochem (San Diego, CA). A polyclonal lectin-type oxidized LDL receptor- (LOX-) 1 antibody was obtained from Biorbyt (San Francisco, CA, USA). A TRIzol reagent was purchased from Invitrogen (Carlsbad, CA, USA).

### 2.2. Animal Feeding and LDL Preparations

Sixteen-week-old male New Zealand White rabbits (~2 kg) were allowed an acclimation period of 2 weeks and then were fed an atherogenic diet (chow supplemented with 5% lard and 0.25% cholesterol) for 2 months, after which blood was drawn from an ear vein and collected in tubes containing EDTA. Plasma was obtained by centrifugation of the pooled blood at 1400 g and 4°C for 10 min. LDL (*d* = 1.019–1.063 g/ml) was isolated by sequential ultracentrifugation from the plasma as described previously [21]. Isolated LDL was then resolved into nLDL and LDL(-) by anion-exchange chromatography on a fast protein liquid chromatographic system (AKTA Explorer; GE, Uppsala, Sweden) as described previously [5, 21], and levels of electronegativity were ascertained through agarose gel electrophoresis using the Beckman Paragon System and were performed according to the manufacturer's instructions [27]. All lipoprotein isolations were carried out within 5 days after the blood was obtained. Cu-ox-LDL was prepared by incubating nLDL with 10 *μ*M CuSO_4_ for 24 h; the reaction was stopped by the addition of EDTA and then dialyzed against 2000 volumes of PBS overnight [27]. Precautions were taken to prevent all LDL preparations from endotoxin contamination and further oxidation [5, 21]. The degree of lipid peroxidation in LDL was determined by measuring thiobarbituric acid-reactive substances (TBARS) using a commercial kit (Cayman, Ann Arbor, MI, USA) according to the manufacturer's protocol. Malondialdehyde (MDA) was used as a standard. Protein concentrations were estimated by the Bradford method (DC Protein Assay Reagent, Bio-Rad, Hercules, CA, USA).

### 2.3. Cell Culture and Lipoprotein Treatment

The THP-1, a human monocytic leukemia cell line, was obtained from the American Type Culture Collection (ATCC, Manassas, VA) and maintained in RPMI 1640 containing 10% FBS as described previously [5, 21]. In these experiments, 2 × 10^5^ cells/well were seeded on 24-well plates and induced differentiation into macrophages by being cultured for 3 days with a medium containing 160 nM PMA. Cells were then cultured in a serum-free RPMI culture medium and treated with 10~40 *μ*g/ml nLDL, LDL(-), or Cu-ox-LDL for 24 h or as indicated. Control cells were treated with phosphate-buffered saline (PBS) in all experiments or as indicated. The culture medium was collected, and levels of cytokines in the medium were determined.

### 2.4. Quantification of IL-1*β*, IL-6, and TNF-*α* in the Culture Medium

Levels of IL-1*β*, IL-6, and TNF-*α* in the culture medium were analyzed by ELISA kits (R&D Systems). All assays were performed according to the manufacturer's instructions.

### 2.5. Western Blot Analysis

Cells were washed with cold PBS and then lysed in RIPA buffer (150 mM NaCl, 50 mM Tris-HCl at pH 7.8, 5 mM EDTA at pH 8.0, 0.5% NP-40, 0.5% Triton X-100, 0.1% sodium dodecyl sulfate (SDS), 1 mM NaF, 1 mM PMSF, 1 mM Na_3_VO_4_, and 1x protease inhibitor cocktail). Protein concentrations were determined by the Bradford method. Proteins (20 *μ*g/well) were loaded and separated on SDS-polyacrylamide gel electrophoresis (PAGE) and transferred to a polyvinylidene difluoride membrane. Levels of ERK1/2, I*κ*B, JNK, p38, p65, phospho-ERK1/2, phospho-I*κ*B, phospho-JNK, phospho-p38, phospho-p65, LOX-1, and *β*-actin were detected using specific antibodies. Bound antibodies were detected using a luminescence imaging system (Fujifilm LAS 4000, Tokyo, Japan). Protein levels on Western blots were quantified using ImageJ software (National Institutes of Health, Bethesda, MD, USA).

### 2.6. Statistical Analysis

Results are shown as the mean ± standard deviation (SD) or standard error (SE). Differences between means were evaluated using Student's *t*-test or by one-way ANOVA followed by Tukey's multiple comparison test and were considered significant at *p* < 0.05.

## 3. Results

### 3.1. LDL(-) Induced Production of IL-1*β*, IL-6, and TNF-*α* by Macrophages

**Figure 1:**
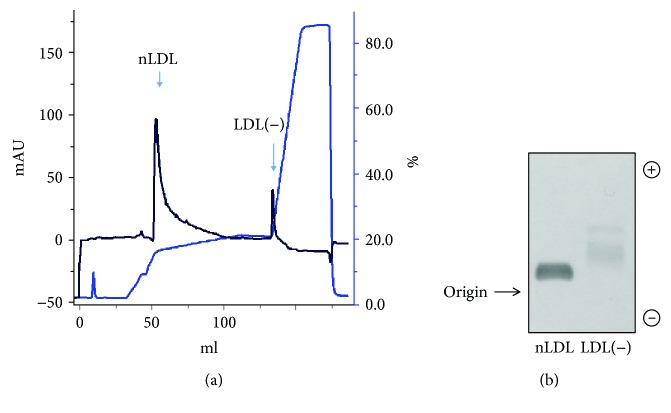
Characterization of LDL(-) in rabbit plasma. Rabbits were fed an atherogenic diet for 6 weeks, then LDL was isolated by ultracentrifugation and loaded onto a UnoQ6 column to separate native (n)LDL and electronegative LDL (LDL(-)). (a) Representative fast protein liquid chromatographic analysis showing the distribution of the nLDL and LDL(-). (b) Isolated nLDL and LDL(-) were analyzed by agarose gel electrophoresis.

**Figure 2:**
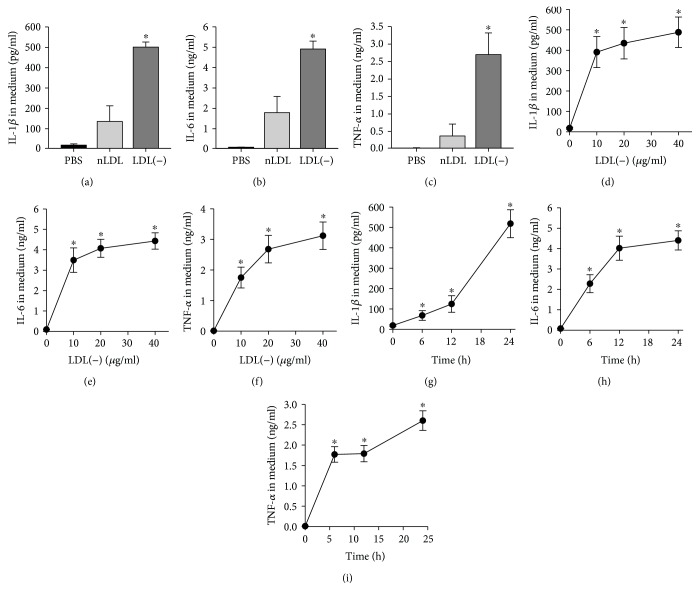
Effects of nLDL and LDL(-) on production of IL-1*β*, IL-6, and TNF-*α* in THP-1 macrophages. THP-1 macrophages were incubated with 20 *μ*g/ml of nLDL or LDL(-) for 24 h, then levels of IL-1*β* (a), IL-6 (b), and TNF-*α* (c) in the medium were measured by ELISA. Values are the mean ± SD of five independent experiments. Differences between means were evaluated using Student's *t*-test. ^∗^*p* < 0.05, compared to PBS- and nLDL-treated cells. Cells were incubated with 0, 10, 20, or 40 *μ*g/ml of LDL(-) for 24 h (d–f) or incubated with 20 *μ*g/ml of LDL(-) for 0, 6, 12, or 24 h (g–i). Then, levels of IL-1*β* (d, g), IL-6 (e, h), and TNF-*α* (f, i) in the medium were determined by ELISA. Values are the mean ± SE of five (in (d–f)) or four (in (g–i)) independent experiments. Data was analyzed by one-way ANOVA followed by Tukey's multiple comparison test. ^∗^*p* < 0.001, compared to 0 *μ*g/ml of LDL(-) or 0 h.

Rabbit plasma total and LDL cholesterol were 56.0 ± 11.0 and 19.5 ± 4.2 mg/dl, respectively, and LDL(-) were not detected in the anion-exchange chromatography in rabbits feeding with the control chow diet. Plasma total cholesterol (C) and LDL-C were 172.1 ± 46.2 and 98.9 ± 23.8 mg/dl, respectively, at 4 weeks and were 342.7 ± 44.5 and 183.1 ± 35.6 mg/dl, respectively, at 12 weeks after feeding with the atherogenic diet. LDL(-) was isolated from plasma of 2 to 3 rabbits each time, and LDL(-) accounted for about 17.2 ± 5.5% of the LDL fraction (ranging from 10 to 27%) (Figure 1(a)). Agarose gel electrophoresis confirmed the electronegativity of rabbit LDL(-) (Figure 1(b)). To examine the effects of nLDL and LDL(-) on inflammatory cytokine production by macrophages, THP-1 macrophages were treated with 20 *μ*g/ml nLDL or LDL(-) for 24 h, and then levels of IL-1*β*, IL-6, and TNF-*α* proteins in culture media were determined. There were negligible levels of IL-1*β*, IL-6, and TNF-*α* in the control cells. Treatment with LDL(-) led to 3.7-, 2.7-, and 7.2-fold increases in IL-1*β*, IL-6, and TNF-*α* production, respectively, compared to treatment with nLDL (Figures 2(a)–2(c)). Treating THP-1 macrophages with LDL(-) (10, 20, and 40 *μ*g/ml) for 24 h induced dose-dependent increases in IL-1*β*, IL-6, and TNF-*α* (Figures 2(d)–2(f)). In addition, treatment with LDL(-) (20 *μ*g/ml) for 6~24 h induced time-dependent increases in IL-1*β*, IL-6, and TNF-*α*, and all had achieved a significant increase after 6 h (Figures 2(g)–2(i)).

### 3.2. LDL(-) Induced Activation of NF-*κ*B and Expressions of NF-*κ*B Downstream Genes

**Figure 3:**
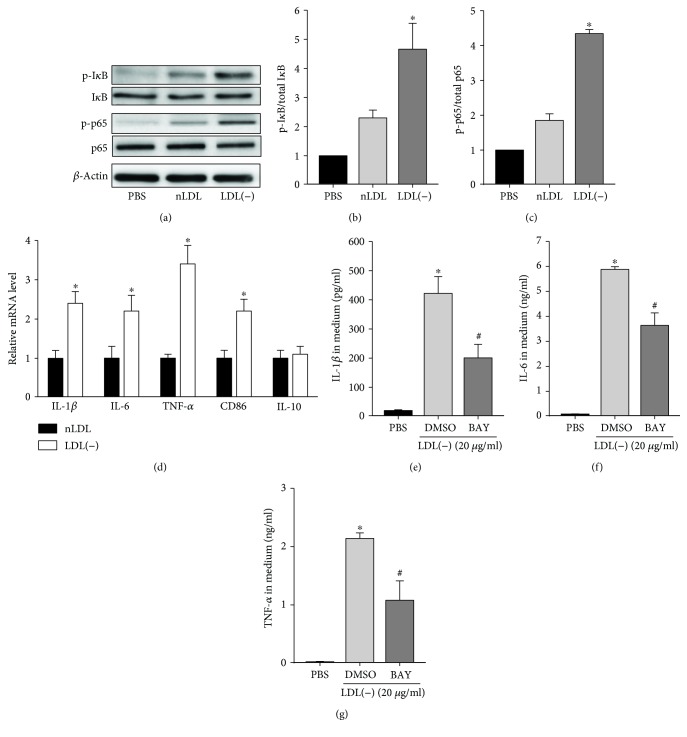
LDL(-)-induced activation of NF-*κ*B and expressions of NF-*κ*B downstream genes. (a) THP-1 macrophages were incubated with 20 *μ*g/ml of native (n)LDL or LDL(-) for 2 h, and then protein levels of total and phosphorylated I*κ*B (I*κ*B and p-I*κ*B, respectively) and p65 (p65 and p-p65, respectively) were determined by Western blotting. *β*-Actin was used as a loading control. (b, c) Relative levels of p-I*κ*B/total I*κ*B (b) and p-p65/total p65 (c) were expressed relative to the control (PBS, relative value = 1). Values are the mean ± SD of three independent experiments. ^∗^*p* < 0.05, compared to PBS- and nLDL-treated cells. (d) THP-1 macrophages were treated with 20 *μ*g/ml of nLDL or LDL(-) for 6 h, and levels of IL-1*β*, IL-6, TNF-*α*, CD86, and IL-10 mRNAs were determined by an RT-qPCR, normalized to levels of GAPDH mRNA, and expressed relative to levels in nLDL-treated cells (relative value = 1). ^∗^*p* < 0.05, compared to nLDL-treated cells. (e–g) Cells were pretreated with 10 *μ*M BAY 11-7082 for 1 h and then treated with LDL(-) (20 *μ*g/ml) for 24 h. Levels of IL-1*β* (e), IL-6 (f), and TNF-*α* (g) in the medium were determined by ELISA. ^∗^*p* < 0.05, compared to DMSO-treated cells.

In a previous study, we demonstrated that STEMI LDL(-) induced activation of NF-*κ*B in macrophages [5]. To investigate whether rabbit LDL(-) is able to induce the activation of NF-*κ*B and expressions of IL-1*β*, IL-6, and TNF-*α* messenger RNAs (mRNAs), macrophages were treated with 20 *μ*g/ml nLDL or LDL(-). Then, protein levels of total and phospho-I*κ*B were determined by Western blotting, and levels of IL-1*β*, IL-6, TNF-*α*, CD86, and IL-10 mRNAs were determined by a quantitative reverse-transcription polymerase chain reaction (RT-qPCR). Figures 3(a)–3(c) shows that levels of phospho-I*κ*B and phospho-p65 were slightly induced by nLDL but were greatly induced by LDL(-) at 2 h. In addition, LDL(-) induced 2.4-, 2.2-, 3.4-, and 2.2-fold increases in IL-1*β*, IL-6, TNF-*α*, and CD86 mRNA levels, respectively, compared to nLDL-treated cells (Figure 3(d)). CD86 is a marker for M1 (classically activated) macrophages; the result suggests that LDL(-) induced THP-1 polarized toward a proinflammatory type. However, the levels of the anti-inflammatory cytokine IL-10 mRNA were about the same in the nLDL- and LDL(-)-treated cells. We then tested if LDL(-)-induced proinflammatory cytokines could be inhibited by an NF-*κ*B inhibitor, BAY 11-7082. Results showed that BAY 11-7082 significantly inhibited LDL(-)-induced IL-1*β*, IL-6, and TNF-*α* (Figures 3(e)–3(g)).

### 3.3. LDL(-)-Induced IL-1*β*, IL-6, and TNF-*α* Production via a LOX-1-Dependent Pathway

**Figure 4:**
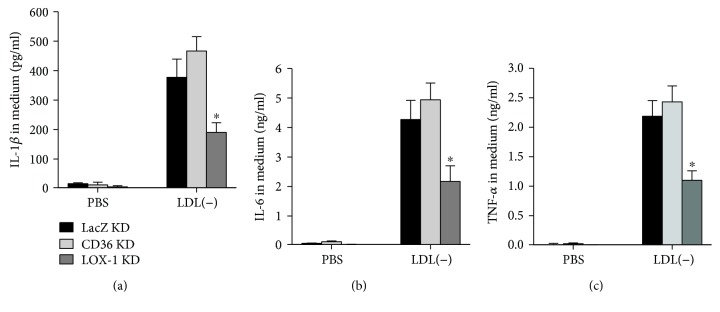
Knockdown of lectin-type oxidized LDL receptor (LOX-1) decreased LDL(-)-induced production of IL-1*β*, IL-6, and TNF-*α*. LOX-1-, CD36-, or LacZ-knockdown (KD) cells were generated as described previously [5, 21]. Knockdown cells were incubated with PBS or 20 mg/ml LDL(-) for 24 h. Levels of IL-1*β* (a), IL-6 (b), and TNF-*α* (c) in the medium were determined. Values are the mean ± SD of three independent experiments. ^∗^*p* < 0.05, compared to CD36- or LacZ-KD cells.

LOX-1 and CD36 are considered major receptors for mildly oxidized LDL [28]. The roles of LOX-1 and CD36 in LDL(-)-induced IL-1*β*, IL-6, and TNF-*α* expressions were investigated using LOX-1- and CD36-knockdown cells, with LacZ-knockdown cells used as a knockdown control. Knockdown cells were generated as described in previous studies [5, 21]. Figures 4(a)–4(c) shows that levels of IL-1*β*, IL-6, and TNF-*α* were low in control LOX-1-, CD36-, and LacZ-knockdown cells. LDL(-) induced IL-1*β*, IL-6, and TNF-*α* to similar levels in LacZ- and CD36-knockdown cells; however, LDL(-)-induced IL-1*β*, IL-6, and TNF-*α* were significantly lower in LOX-1-knockdown cells. These results suggest that LDL(-) induced IL-1*β*, IL-6, and TNF-*α* through a LOX-1-dependent pathway in macrophages.

### 3.4. LDL(-) Is More Potent than Cu-ox-LDL in Inducing Production of IL-1*β*, IL-6, and TNF-*α* and Activation of NF-*κ*B and MAPKs in Macrophages

**Figure 5:**
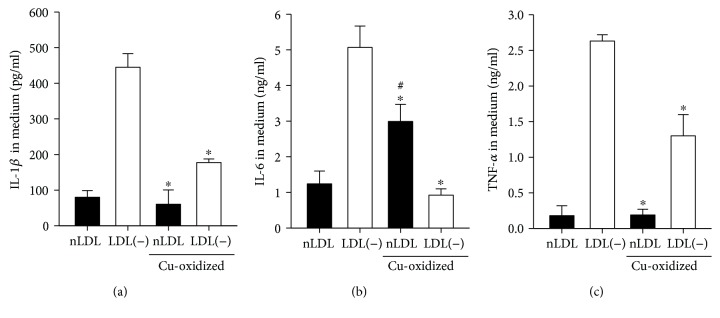
Effects of copper oxidation of nLDL and LDL(-) on IL-1*β*, IL-6, and TNF-*α* production in THP-1 macrophages. THP-1 macrophages were treated with 20 *μ*g/ml of nLDL, LDL(-), Cu-ox-nLDL, or Cu-ox-LDL(-) for 24 h, then levels of IL-1*β* (a), IL-6 (b), and TNF-*α* (c) in the medium were measured by ELISA. Values are the mean ± SD of three independent experiments. ^∗^*p* < 0.05, compared to LDL(-)-treated cells.

LOX-1 has been shown to have a higher reactivity with mildly ox-LDL, such as LDL(-), than with extensively oxidized LDL [29]. We then compared the proinflammatory effects of LDL(-) and extensively ox-LDL. To prepare extensively ox-LDL, nLDL and LDL(-) were incubated with copper for 24 h to, respectively, produce Cu-ox-nLDL and Cu-ox-LDL(-). The degrees of oxidation were estimated by measuring TBARS. Levels of TBARS with nLDL and LDL(-) were both <1 nmol/mg protein, while levels of TBARS with Cu-ox-nLDL and Cu-ox-LDL(-) were 17.6 and 15.8 nmol/mg protein, respectively. Then, 20 *μ*g/ml nLDL, LDL(-), Cu-ox-nLDL, and Cu-ox-LDL(-) were incubated with THP-1 macrophages for 24 h.  Figure 5 shows that Cu-ox-nLDL induced higher levels of IL-6 than nLDL but not the levels of IL-1*β* and TNF-*α*. However, oxidation of LDL(-) resulted in decrease of its ability to induce IL-1*β*, IL-6, and TNF-*α*. Cu-ox-LDL(-) induced lower levels of IL-1*β*, IL-6, and TNF-*α* than did LDL(-). Further, LDL(-) induced significantly higher levels of IL-1*β*, IL-6, and TNF-*α* than did either nLDL or Cu-ox-nLDL (Figure 5). These results show that LDL(-) is more potent in inducing IL-1*β*, IL-6, and TNF-*α* than nLDL and extensively ox-LDL.

### 3.5. Effects of LDL(-) and Cu-ox-nLDL on Phosphorylation of I*κ*B, p38, ERK1/2, and JNK

**Figure 6:**
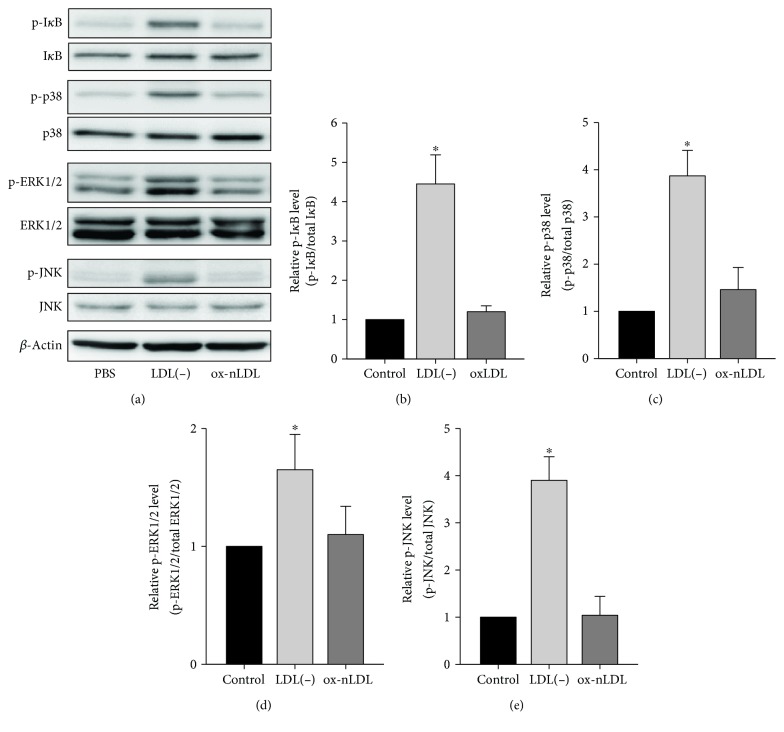
Effects of LDL(-) and Cu-ox-nLDL on the activation of I*κ*B, p38, ERK1/2, and JNK in THP-1 macrophages. (a) Cells were treated with 20 *μ*g/ml of LDL(-) or Cu-ox-nLDL for 2 h, and then levels of phosphorylated I*κ*B (p-I*κ*B), total I*κ*B, p-p38, total p38, p-ERK1/2, total ERK1/2, p-JNK, and total JNK were determined by Western blotting. *β*-Actin was used as a loading control. (b–e) Protein levels were quantified using ImageJ software, and relative levels of p-I*κ*B/total I*κ*B (b), p-p38/total p38 (c), p-ERK1/2/total ERK1/2 (d), and p-JNK/total JNK (e) are expressed relative to PBS-treated cells (relative level = 1). Values are the mean ± SD of three independent experiments. ^∗^*p* < 0.05, compared to corresponding PBS-treated and Cu-ox-nLDL-treated cells.

**Figure 7:**
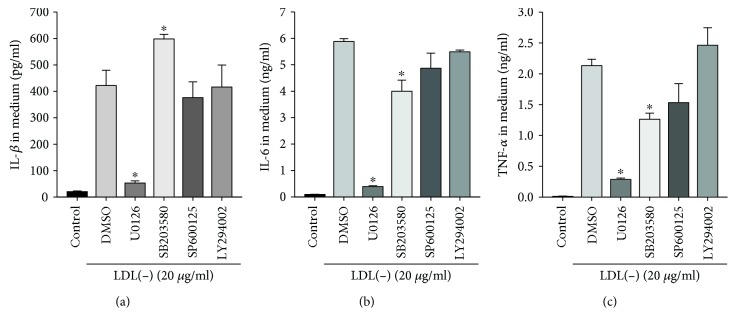
Effects of MAPK inhibitors on the LDL(-)-induced IL-1*β*, IL-6, and TNF-*α* in macrophages. Cells were preincubated with DMSO, U0126 (10 *μ*M), SB203580 (10 *μ*M), SP600125 (15 *μ*M), or LY294002 (10 *μ*M) for 1 h, then treated with LDL(-) for 24 h. Levels of IL-1*β* (a), IL-6 (b), and TNF-*α* (c) in the medium were determined by ELISA. Values are the mean ± SD of three independent experiments. ^∗^*p* < 0.05, compared to DMSO-treated cells.

Next, we compared the effects of LDL(-) and Cu-ox-nLDL (20 *μ*g/ml) on inducing levels of phosphorylated I*κ*B and MAPKs, including p38, ERK1/2, and JNK.  Figure 6 shows that the levels of phosphorylated I*κ*B, MAPK-p38, ERK1/2, and JNK were all greatly induced by LDL(-) but the levels were not induced by Cu-ox-nLDL. To examine the involvement of MAPKs in LDL(-) induction of IL-1*β*, IL-6, and TNF-*α* in THP-1 macrophages, cells were pretreated for 30 min with 10 mM of U0126, 50 mM of LY294002, 20 mM of SB203580, 0.5 mM of L-JNKi 1 trifluoroacetate, or DMSO (vehicle). Subsequently, 20 mg/ml of LDL(-) was added and incubated for 24 h.  Figure 7 shows that LDL(-)-induced IL-1*β*, IL-6, and TNF-*α* were all inhibited by U0126; in addition, LDL(-)-induced IL-6 and TNF-*α* were moderately inhibited by SB203580. These results suggest that LDL(-)-induced IL-1*β* occurs through an ERK1/2-dependent pathway and induction of IL-6 and TNF-*α* occurs through p38- and ERK1/2-dependent pathways.

## 4. Discussion

In this study, we showed that an atherogenic diet induced generation of LDL(-), a type of circulating ox-LDL, in rabbits. We also demonstrated that LDL(-) is potent in inducing activation of NF-*κ*B and MAPK signaling pathways and production of IL-1*β*, IL-6, and TNF-*α* in THP-1 macrophages. IL-1*β* is a major proinflammatory cytokine in the pathogenesis of cardiovascular diseases. Knockout IL-1*β* in atherosclerosis-prone ApoE-deficient mice leads to attenuation of atherosclerosis development [30]. Moreover, blocking IL-1*β* with a monoclonal antibody, canakinumab, has resulted in a lower rate of recurrent cardiovascular events, cardiovascular complications, and cardiovascular mortality in patients with MI [31]. IL-6 is a pleiotropic cytokine that has been shown to contribute to atherosclerotic plaque development and plaque destabilization [32] and induction of the hepatic acute phase response protein such as C-reactive protein (CRP) [33], increasing the expression of ICAM-1 in endothelial cells [34]. TNF-*α* was originally identified as a circulating factor which can cause necrosis of tumors [35] and was also later found crucially involved in the pathogenesis and progression of atherosclerosis [36]. TNF-*α* induces expression of adhesion molecules, proinflammatory cytokines, and chemokine receptors in endothelial cells [37]. Therefore, induction of IL-1*β*, IL-6, and TNF-*α* by LDL(-) in macrophages has a link between LDL(-) and inflammation in hypercholesterolemia. In addition, higher levels of LDL(-) and inflammation were shown in hypercholesterolemic patients than in normolipidemic subjects [38–42]. Supplementary [Supplementary-material supplementary-material-1] shows that rabbit LDL(-) induced the granulocyte colony-stimulating factor (G-CSF) to a similar level as that induced by STEMI LDL(-) [21]. These properties revealed that LDL(-) is a highly atherogenic lipoprotein and imply a pathogenic role for LDL(-) in hypercholesterolemia.

Atherosclerosis is now considered a chronic inflammatory disorder [3, 4, 43]. Plasma levels of the proinflammatory cytokines IL-1*β*, IL-6, and TNF-*α* were higher in atherosclerotic patients than in normal subjects [44]. Moreover, an increased level of LDL(-) was associated with high cardiovascular risk [45, 46]. Our data showed that LDL(-) induced activation of NF-*κ*B and MAPKs and the subsequent production of proinflammatory cytokines in macrophages; hence, LDL(-)-activated macrophages may be associated with inflammation in atherosclerosis. Our preliminary studies showed that replacing the atherogenic diet with normal chow would decrease levels of both LDL and LDL(-) in rabbits and hamsters. Clinical studies demonstrated that statin therapy results in a progressive decrease in the proportion of LDL(-) and attenuates inflammation in hyperlipidemic patients [38, 40, 47, 48]. Such outcomes could be due to the cholesterol-lowering and/or anti-inflammatory effects of statins. These results suggest that inflammation is associated with hypercholesterolemia and lowering cholesterol by dietary control or statins may have beneficial effects on reducing LDL(-) and inflammation.

LOX-1 was implicated in vascular inflammation and the pathogenesis of atherosclerosis [49]. Overexpression of LOX-1 in ApoE null mice (LOX-1tg/ApoE^–/–^) increased macrophage infiltration and enhanced expressions of intracellular adhesion molecule- (ICAM-) 1 and vascular cell adhesion molecule- (VCAM-) 1 and accumulation of ox-LDL in coronary arteries [50]. Specific overexpression of LOX-1 in the endothelium also promoted atherosclerosis and inflammation in ApoE null mice [51], whereas LOX-1-knockout reduced atherosclerotic lesions and proinflammatory signals in LDL receptor- (LDLR-) null mice fed an atherogenic diet [52]. Those results indicate that LOX-1 plays a critical role in the pathogenesis of atherosclerosis. We recently reported that LDL(-) from STEMI patients induced production of IL-1*β*, G-CSF, and GM-CSF through a LOX-1-dependent pathway [5, 21]. Similar to human LDL(-), rabbit LDL(-) induced IL-1*β*, IL-6, and TNF-*α* through a LOX-1-dependent pathway (Figure 4). Moreover, the LOX-1 protein level was induced by rabbit LDL(-) as it was upregulated by STEMI LDL(-) (Supplementary [Supplementary-material supplementary-material-1]). Kakutani et al. demonstrated that copper-oxidized rabbit LDL is a ligand for LOX-1 [29]. Those authors further demonstrated that mildly oxidized human or rabbit LDL had higher reactivity to LOX-1 than did either less- or more-oxidized LDL. Those results also supported that rabbit LDL(-) induced an inflammatory response in macrophages through a LOX-1-dependent pathway and the LDL(-)/LOX-1 axis may play important roles in inflammation and atherogenesis. However, the involvement of other receptors cannot be excluded.

Although extensively oxidized LDL was shown to be able to activate NF-*κ*B and elicit proinflammatory cytokines to various extents [53–55], comparing our results to results from other studies is difficult, because the source and degree of oxidation of the ox-LDL used in different studies might not be the same. In this study, we used relatively smaller amounts (20 *μ*g/ml) of LDL(-) and Cu-ox-LDL compared to larger amounts (50 to 200 *μ*g/ml) of extensively oxidized LDL used in other studies [53–56]. Moreover, results of this study comparing LDL(-), Cu-ox-nLDL, and Cu-ox-LDL(-) clearly showed that LDL(-) was more proinflammatory than was extensively oxidized LDL. Several different receptors, including scavenger receptors-AI and II (SR-AI and II), CD36, and LOX-1, have been identified to recognize ox-LDL and mediate ox-LDL-cellular interactions. The specificity of the different receptors to ox-LDL with the degree of oxidation is different [28]. SR-AI and II are considered to be the most specific for extensively oxidized LDL [57]; CD36 was shown to bind and internalize extensively or moderately oxidized LDL [58], while LOX-1 has high specificity to moderately oxidized LDL [29]. Activation of LOX-1 has been shown to induce several intracellular signaling pathways, including MAPKs, protein kinase C, and transcriptional factors NF-*κ*B and AP-1. Our previous study showed that STEMI LDL(-) induced higher levels of G-CSF and GM-CSF than did extensively Cu-ox-nLDL or Cu-ox-LDL(-) in macrophages [21]. LDL(-) is considered a moderately oxidized LDL and thus support LDL(-) being more potent in inducing the production of proinflammatory cytokines than extensively oxidized LDL.

Our results showed that LDL(-) induced activation of ERK1/2, JNK, and p38 MAPK and showed that LDL(-)-induced IL-1*β*, IL-6, and TNF-*α* can be inhibited by U0126, a MEK inhibitor, and LDL(-)-induced IL-6 and TNF-*α* can be partially inhibited by SB203580, a p38 inhibitor. Estruch et al. have demonstrated that p38 MAPK is involved in LDL(-)-induced activation of NF-*κ*B and AP-1. However, our results showed that LDL(-)-induced IL-1*β* was increased by SB203580; the mechanism underlying this is not clear and requires further investigation. In our previous report, U0126, a MEK inhibitor that inhibited activation of ERK1/2, inhibited LDL(-)-induced phosphorylation of ERK1/2. ERK signaling is known to be associated with various cellular processes, including proliferation, differentiation, and survival, and it was implicated in the pathogenesis of many diseases, including stroke, neurological diseases, and cancer [59, 60]. ERK1/2 can be regulated by mitogens and endotoxins. Upon stimulation, ERK1/2 is activated by phosphorylation of a threonine and tyrosine residue in the motif Thr-Glu-Tyr within the kinase domain [61]. Inhibition of the ERK signaling pathway was shown to inhibit allergic airway inflammation [62] and focal cerebral ischemia [63]. We reported that ERK1/2 is activated by LDL(-) through a LOX-1-dependent pathway and inhibition of the ERK1/2 pathway decreased the release of G-CSF and GM-CSF in STEMI LDL(-)-treated macrophages [21]. Moreover, ERK1/2 have been reported to be activated by ox-LDL in carotid arteries [64] and in human umbilical vein endothelial cells [65] through a LOX-1-dependent manner. In this report, we showed that in macrophages, activation of ERK1/2 is necessary for the LDL(-)-induced production of IL-1*β*, IL-6, and TNF-*α*. Moreover, ERK1/2 was shown to be activated by ox-LDL in vascular smooth muscle cells and endothelial cells via LOX-1 [66–68]. Inhibition of ERK suppressed cell proliferation in ox-LDL-treated vascular smooth muscle cells [66, 67] and reduced matrix metalloproteinase expression in endothelial cells [68]. These results suggest that the LOX-1/ERK axis may serve as a potential therapeutic target for LDL(-)-mediated atherosclerosis.

## 5. Conclusions

In conclusion, our data provide evidence that an atherogenic diet induces highly proinflammatory LDL(-) in rabbits. The LDL(-) was more potent than native or extensively oxidized LDL in stimulating proinflammatory signals and cytokines. Moreover, we also elucidated that LDL(-) triggered production of IL-1*β*, IL-6, and TNF-*α* through a LOX-1/NF-*κ*B/ERK-dependent pathway. These results provide a link between an atherogenic diet and inflammation in the pathogenesis of atherosclerosis.

## Data Availability

The ELISA and Western data used to support the findings of this study are available from the corresponding author upon request.

## Abbreviations

Cu-ox-LDL: Copper-oxidized LDL
ELISA: Enzyme-linked immunosorbent assay
ERK: Extracellular signal-regulated kinase
I*κ*B: Inhibitor of NF-*κ*B
IL: Interleukin
JNK: C-Jun N-terminal kinase
LDL: Low-density lipoprotein
LDL(-): Electronegative LDL
LOX-1: Lectin-type oxidized LDL receptor 1
MAPK: Mitogen-activated protein kinase
NF-*κ*B: Nuclear factor-*κ*B
nLDL: Native LDL
ox-LDL: Oxidized LDL
RT-qPCR: Reverse-transcription quantitative polymerase chain reaction
SDS-PAGE: Sodium dodecyl sulfate polyacrylamide gel electrophoresis
STEMI: ST-elevation myocardial infarction
TNF: Tumor necrosis factor.

## Supplementary Materials

Supplementary Materials Supplementary Figure 1: rabbit electronegative low-density lipoprotein (LDL(-)) induced granulocyte colony-stimulating factor (G-CSF) in THP-1 macrophages. Supplementary Figure 2: LDL(-)-induced lectin-type oxidized LDL receptor (LOX-1) expression in THP-1 cells.

## References

[1] Steinberg D., Parthasarathy S., Carew T. E., Khoo J. C., Witztum J. L. (1989). Beyond cholesterol. Modifications of low-density lipoprotein that increase its atherogenicity. *The New England Journal of Medicine*.

[2] Natarajan V., Scribner W. M., Hart C. M., Parthasarathy S. (1995). Oxidized low density lipoprotein-mediated activation of phospholipase D in smooth muscle cells: a possible role in cell proliferation and atherogenesis. *Journal of Lipid Research*.

[3] Ross R. (1999). Atherosclerosis — an inflammatory disease. *The New England Journal of Medicine*.

[4] Libby P., Ridker P. M., Maseri A. (2002). Inflammation and atherosclerosis. *Circulation*.

[5] Yang T. C., Chang P. Y., Lu S. C. (2017). L5-LDL from ST-elevation myocardial infarction patients induces IL-1*β* production via LOX-1 and NLRP3 inflammasome activation in macrophages. *American Journal of Physiology-Heart and Circulatory Physiology*.

[6] Van Tassell B. W., Toldo S., Mezzaroma E., Abbate A. (2013). Targeting interleukin-1 in heart disease. *Circulation*.

[7] Ridker P. M., Everett B. M., Thuren T. (2017). Antiinflammatory therapy with canakinumab for atherosclerotic disease. *The New England Journal of Medicine*.

[8] de Castellarnau C., Sánchez-Quesada J. L., Benítez S. (2000). Electronegative LDL from normolipemic subjects induces IL-8 and monocyte chemotactic protein secretion by human endothelial cells. *Arteriosclerosis, Thrombosis, and Vascular Biology*.

[9] Benítez S., Bancells C., Ordóñez-Llanos J., Sánchez-Quesada J. L. (2007). Pro-inflammatory action of LDL(−) on mononuclear cells is counteracted by increased IL10 production. *Biochimica et Biophysica Acta (BBA) - Molecular and Cell Biology of Lipids*.

[10] Estruch M., Bancells C., Beloki L., Sanchez-Quesada J. L., Ordóñez-Llanos J., Benitez S. (2013). CD14 and TLR4 mediate cytokine release promoted by electronegative LDL in monocytes. *Atherosclerosis*.

[11] Estruch M., Rajamäki K., Sanchez-Quesada J. L. (2015). Electronegative LDL induces priming and inflammasome activation leading to IL-1*β* release in human monocytes and macrophages. *Biochimica et Biophysica Acta (BBA) - Molecular and Cell Biology of Lipids*.

[12] Ligi D., Benitez S., Croce L. (2018). Electronegative LDL induces MMP-9 and TIMP-1 release in monocytes through CD14 activation: inhibitory effect of glycosaminoglycan sulodexide. *Biochimica et Biophysica Acta - Molecular Basis of Disease*.

[13] Hulthe J., Fagerberg B. (2002). Circulating oxidized LDL is associated with subclinical atherosclerosis development and inflammatory cytokines (AIR study). *Arteriosclerosis, Thrombosis, and Vascular Biology*.

[14] Sanchez-Quesada J. L., Benítez S., Otal C., Franco M., Blanco-Vaca F., Ordóñez-Llanos J. (2002). Density distribution of electronegative LDL in normolipemic and hyperlipemic subjects. *The Journal of Lipid Research*.

[15] Chan H.-C., Ke L. Y., Chu C. S. (2013). Highly electronegative LDL from patients with ST-elevation myocardial infarction triggers platelet activation and aggregation. *Blood*.

[16] Lu J., Jiang W., Yang J. H. (2008). Electronegative LDL impairs vascular endothelial cell integrity in diabetes by disrupting fibroblast growth factor 2 (FGF2) autoregulation. *Diabetes*.

[17] Chang P. Y., Chen Y. J., Chang F. H. (2013). Aspirin protects human coronary artery endothelial cells against atherogenic electronegative LDL via an epigenetic mechanism: a novel cytoprotective role of aspirin in acute myocardial infarction. *Cardiovascular Research*.

[18] Wellen K. E., Hotamisligil G. S. (2005). Inflammation, stress, and diabetes. *The Journal of Clinical Investigation*.

[19] van der Laan A. M., Hirsch A., Robbers L. F. H. J. (2012). A proinflammatory monocyte response is associated with myocardial injury and impaired functional outcome in patients with ST-segment elevation myocardial infarction: monocytes and myocardial infarction. *American Heart Journal*.

[20] Ridker P. M. (2012). Hyperlipidemia as an instigator of inflammation: inaugurating new approaches to vascular prevention. *Journal of the American Heart Association*.

[21] Yang T. C., Chang P. Y., Kuo T. L., Lu S. C. (2017). Electronegative L5-LDL induces the production of G-CSF and GM-CSF in human macrophages through LOX-1 involving NF-*κ*B and ERK2 activation. *Atherosclerosis*.

[22] Chen W. Y., Chen F. Y., Lee A. S. (2015). Sesamol reduces the atherogenicity of electronegative L5 LDL in vivo and in vitro. *Journal of Natural Products*.

[23] Teixeira Damasceno N. R., Apolinário E., Dias Flauzino F., Fernandes I., Abdalla D. S. P. (2007). Soy isoflavones reduce electronegative low-density lipoprotein (LDL^−^) and anti-LDL^−^ autoantibodies in experimental atherosclerosis. *European Journal of Nutrition*.

[24] Buchwald H. (1965). Myocardial infarction in rabbits induced solely by a hypercholesterolemic diet. *Journal of Atherosclerosis Research*.

[25] Fan J., Watanabe T. (2000). Cholesterol-fed and transgenic rabbit models for the study of atherosclerosis. *Journal of Atherosclerosis and Thrombosis*.

[26] Liaw Y. W., Lin C. Y., Lai Y. S. (2014). A vaccine targeted at CETP alleviates high fat and high cholesterol diet-induced atherosclerosis and non-alcoholic steatohepatitis in rabbit. *PLoS One*.

[27] Chang P. Y., Lu S. C., Su T. C. (2004). Lipoprotein-X reduces LDL atherogenicity in primary biliary cirrhosis by preventing LDL oxidation. *Journal of Lipid Research*.

[28] Levitan I., Volkov S., Subbaiah P. V. (2010). Oxidized LDL: diversity, patterns of recognition, and pathophysiology. *Antioxidants & Redox Signaling*.

[29] Kakutani M., Ueda M., Naruko T., Masaki T., Sawamura T. (2001). Accumulation of LOX-1 ligand in plasma and atherosclerotic lesions of Watanabe heritable hyperlipidemic rabbits: identification by a novel enzyme immunoassay. *Biochemical and Biophysical Research Communications*.

[30] Kirii H., Niwa T., Yamada Y. (2003). Lack of interleukin-1*β* decreases the severity of atherosclerosis in ApoE-deficient mice. *Arteriosclerosis, Thrombosis, and Vascular Biology*.

[31] Ridker P. M., MacFadyen J. G., Everett B. M. (2018). Relationship of C-reactive protein reduction to cardiovascular event reduction following treatment with canakinumab: a secondary analysis from the CANTOS randomised controlled trial. *The Lancet*.

[32] Schuett H., Luchtefeld M., Grothusen C., Grote K., Schieffer B. (2009). How much is too much? Interleukin-6 and its signalling in atherosclerosis. *Thrombosis and Haemostasis*.

[33] Castell J. V., Gómez-Lechón M. J., David M., Fabra R., Trullenque R., Heinrich P. C. (1990). Acute-phase response of human hepatocytes: regulation of acute-phase protein synthesis by interleukin-6. *Hepatology*.

[34] Romano M., Sironi M., Toniatti C. (1997). Role of IL-6 and its soluble receptor in induction of chemokines and leukocyte recruitment. *Immunity*.

[35] Carswell E. A., Old L. J., Kassel R. L., Green S., Fiore N., Williamson B. (1975). An endotoxin-induced serum factor that causes necrosis of tumors. *Proceedings of the National Academy of Sciences of the United States of America*.

[36] McKellar G. E., McCarey D. W., Sattar N., McInnes I. B. (2009). Role for TNF in atherosclerosis? Lessons from autoimmune disease. *Nature Reviews Cardiology*.

[37] Zhang H., Park Y., Wu J. (2009). Role of TNF-*α* in vascular dysfunction. *Clinical Science*.

[38] Sanchez-Quesada J. L., Otal-Entraigas C., Franco M. (1999). Effect of *simvastatin* treatment on the electronegative low-density lipoprotein present in patients with heterozygous familial hypercholesterolemia. *The American Journal of Cardiology*.

[39] Chen H. H., Hosken B. D., Huang M. (2007). Electronegative LDLs from familial hypercholesterolemic patients are physicochemically heterogeneous but uniformly proapoptotic. *Journal of Lipid Research*.

[40] Chu C.-S., Wang Y. C., Lu L. S. (2013). Electronegative low-density lipoprotein increases C-reactive protein expression in vascular endothelial cells through the LOX-1 receptor. *PLoS One*.

[41] Holven K. B., Narverud I., Lindvig H. W. (2014). Subjects with familial hypercholesterolemia are characterized by an inflammatory phenotype despite long-term intensive cholesterol lowering treatment. *Atherosclerosis*.

[42] Narverud I., Halvorsen B., Nenseter M. S. (2013). Oxidized LDL level is related to gene expression of tumour necrosis factor super family members in children and young adults with familial hypercholesterolaemia. *Journal of Internal Medicine*.

[43] Raggi P., Genest J., Giles J. T. (2018). Role of inflammation in the pathogenesis of atherosclerosis and therapeutic interventions. *Atherosclerosis*.

[44] Haddy N., Sass C., Droesch S. (2003). IL-6, TNF-*α* and atherosclerosis risk indicators in a healthy family population: the STANISLAS cohort. *Atherosclerosis*.

[45] Akyol S., Lu J., Akyol O. (2017). The role of electronegative low-density lipoprotein in cardiovascular diseases and its therapeutic implications. *Trends in Cardiovascular Medicine*.

[46] Sánchez-Quesada J. L., Benítez S., Ordóñez-Llanos J. (2004). Electronegative low-density lipoprotein. *Current Opinion in Lipidology*.

[47] Zhang B., Miura S. I., Yanagi D. (2008). Reduction of charge-modified LDL by statin therapy in patients with CHD or CHD risk factors and elevated LDL-C levels: the SPECIAL study. *Atherosclerosis*.

[48] Zhang B., Matsunaga A., Rainwater D. L. (2009). Effects of rosuvastatin on electronegative LDL as characterized by capillary isotachophoresis: the ROSARY study. *Journal of Lipid Research*.

[49] Pirillo A., Norata G. D., Catapano A. L. (2013). LOX-1, OxLDL, and atherosclerosis. *Mediators of Inflammation*.

[50] Inoue K., Arai Y., Kurihara H., Kita T., Sawamura T. (2005). Overexpression of lectin-like oxidized low-density lipoprotein receptor-1 induces intramyocardial vasculopathy in apolipoprotein E–null mice. *Circulation Research*.

[51] Akhmedov A., Rozenberg I., Paneni F. (2014). Endothelial overexpression of *LOX-1* increases plaque formation and promotes atherosclerosis in vivo. *European Heart Journal*.

[52] Mehta J. L., Sanada N., Hu C. P. (2007). Deletion of LOX-1 reduces atherogenesis in LDLR knockout mice fed high cholesterol diet. *Circulation Research*.

[53] Liu W., Yin Y., Zhou Z., He M., Dai Y. (2014). OxLDL-induced IL-1beta secretion promoting foam cells formation was mainly via CD36 mediated ROS production leading to NLRP3 inflammasome activation. *Inflammation Research*.

[54] Zhang H., Zhai Z., Zhou H. (2015). Puerarin inhibits oxLDL-induced macrophage activation and foam cell formation in human THP1 macrophage. *BioMed Research International*.

[55] Schwarz A., Bonaterra G. A., Schwarzbach H., Kinscherf R. (2017). Oxidized LDL-induced JAB1 influences NF-*κ*B independent inflammatory signaling in human macrophages during foam cell formation. *Journal of Biomedical Science*.

[56] Jiang Y., Wang M., Huang K. (2012). Oxidized low-density lipoprotein induces secretion of interleukin-1*β* by macrophages via reactive oxygen species-dependent NLRP3 inflammasome activation. *Biochemical and Biophysical Research Communications*.

[57] Steinbrecher U. P. (1987). Oxidation of human low density lipoprotein results in derivatization of lysine residues of apolipoprotein B by lipid peroxide decomposition products. *Journal of Biological Chemistry*.

[58] Kunjathoor V. V., Febbraio M., Podrez E. A. (2002). Scavenger receptors class A-I/II and CD36 are the principal receptors responsible for the uptake of modified low density lipoprotein leading to lipid loading in macrophages. *Journal of Biological Chemistry*.

[59] Roskoski R. (2012). ERK1/2 MAP kinases: structure, function, and regulation. *Pharmacological Research*.

[60] Wortzel I., Seger R. (2011). The ERK cascade: distinct functions within various subcellular organelles. *Genes & Cancer*.

[61] Wainstein E., Seger R. (2016). The dynamic subcellular localization of ERK: mechanisms of translocation and role in various organelles. *Current Opinion in Cell Biology*.

[62] Duan W., Chan J. H. P., Wong C. H., Leung B. P., Wong W. S. F. (2004). Anti-inflammatory effects of mitogen-activated protein kinase kinase inhibitor U0126 in an asthma mouse model. *The Journal of Immunology*.

[63] Wang Z. Q., Wu D. C., Huang F. P., Yang G. Y. (2004). Inhibition of MEK/ERK 1/2 pathway reduces pro-inflammatory cytokine interleukin-1 expression in focal cerebral ischemia. *Brain Research*.

[64] Akhmedov A., Camici G. G., Reiner M. F. (2017). Endothelial LOX-1 activation differentially regulates arterial thrombus formation depending on oxLDL levels: role of the Oct-1/SIRT1 and ERK1/2 pathways. *Cardiovascular Research*.

[65] Thakkar S., Wang X., Khaidakov M. (2015). Structure-based design targeted at LOX-1, a receptor for oxidized low-density lipoprotein. *Scientific Reports*.

[66] Zhang Z., Zhang M., Li Y. (2013). Simvastatin inhibits the additive activation of ERK1/2 and proliferation of rat vascular smooth muscle cells induced by combined mechanical stress and oxLDL through LOX-1 pathway. *Cellular Signalling*.

[67] Zhang Z., Zhang D., Du B., Chen Z. (2017). Hyperoside inhibits the effects induced by oxidized low-density lipoprotein in vascular smooth muscle cells via oxLDL-LOX-1-ERK pathway. *Molecular and Cellular Biochemistry*.

[68] Tsai K. L., Chang Y. L., Huang P. H. (2016). *Ginkgo biloba* extract inhibits oxidized low-density lipoprotein (oxLDL)-induced matrix metalloproteinase activation by the modulation of the lectin-like oxLDL receptor 1-regulated signaling pathway in human umbilical vein endothelial cells. *Journal of Vascular Surgery*.

