# Supplementary material for: Electronegative LDL from Rabbits Fed with Atherogenic Diet Is Highly Proinflammatory
Source: Mediators Inflamm. 2019 Aug 22;2019:6163130. doi: 10.1155/2019/6163130 (PMC6724430; doi:10.1155/2019/6163130)
Supplement: Supplementary Materials — Supplementary Figure 1: rabbit electronegative low-density lipoprotein (LDL(-)) induced granulocyte colony-stimulating factor (G-CSF) in THP-1 macrophages. Supplementary Figure 2: LDL(-)-induced lectin-type oxidized LDL receptor (LOX-1) expression in THP-1 cells. [file 6163130.f1.pdf]

Supplementary Figures

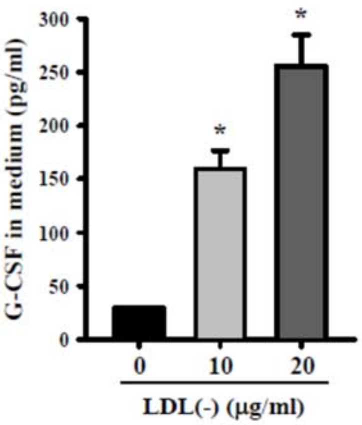

Supplementary Figure 1. Rabbit electronegative low-density lipoprotein (LDL(-)) induced granulocyte colony-stimulating factor (G-CSF) in THP-1 macrophages. THP-1 macrophages were incubated with 0, 10, or 20 μg/mL rabbit LDL(-) for 24 h. Levels of G-CSF in the medium were determined by an ELISA. Values are the mean ± SD of three independent experiments. * *p*<0.05, compared to PBS-treated (0 mg/ml of LDL(-)) cells.

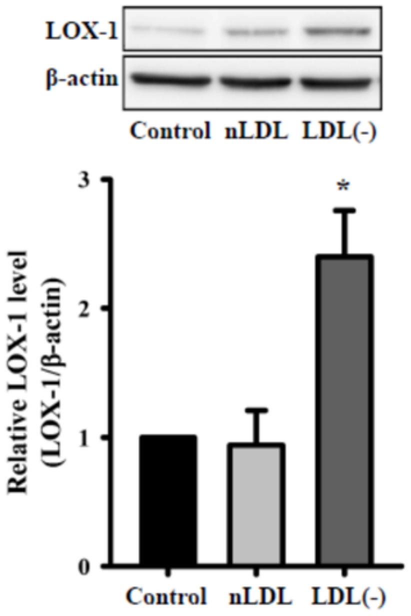

Supplementary Figure 2. LDL(-)-induced lectin-type oxidized LDL receptor (LOX-1) expression in THP-1 cells. THP-1 macrophages were incubated with 20 μg/mL rabbit LDL(-) or nLDL for 24 h. The protein levels of LOX-1 were determined by Western blot and were quantified by imaged J. Relative levels of LOX-1/β-actin were expressed relative to the untreated control (relative value = 1). The values are mean ± S.E. of three independent experiments. *$p<0.05$, compared to the control and nLDL.

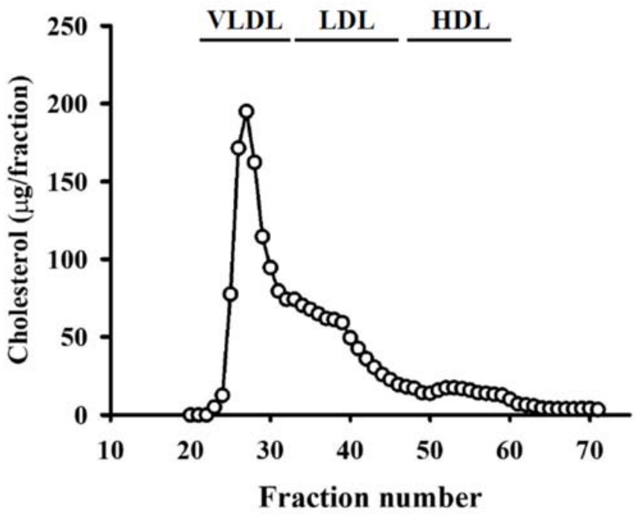

Supplementary Figure 3. Gel filtration chromatography of lipoproteins by gel filtration chromatography. Pooled plasma form 3 rabbits (1 ml) were applied to a Superose 6HR 10/30 column at a flow rate of 0.25 ml/min and collected 0.5 ml/tube. Cholesterol concentrations in each fraction were measured using enzymatic assay kits (Randox, Crumlin, UK).

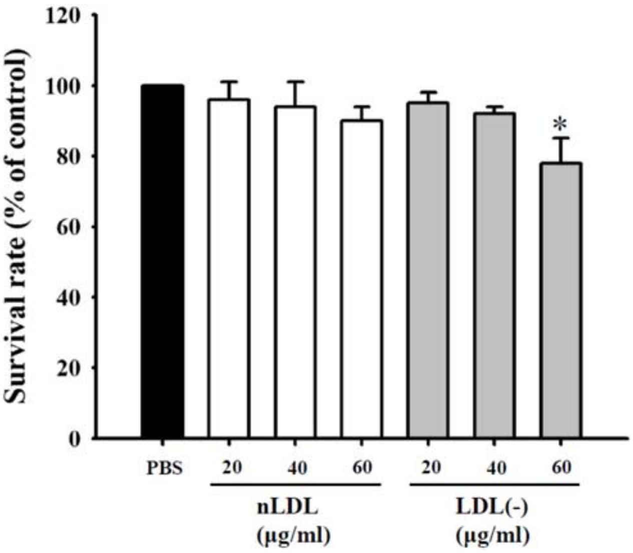

Supplementary Figure 4. Cell viability of THP-1 macrophages treated with nLDL and LDL(-). THP-1 macrophages were incubated with 20, 40 or 60 μg/mL LDL(-) for 24 h, then cell viability was determined by MTT assay. *$p < 0.05$, compared to the control cells.

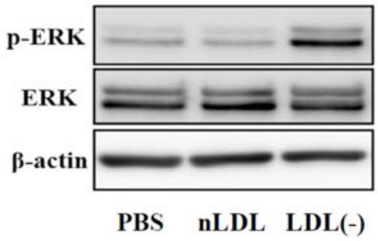

Supplementary Figure 5. Effects of nLDL and LDL(-) on the activation of ERK1/2 in THP-1 macrophages. Cells were treated with 20 μg/ml of nLDL or LDL(-)for 2 h, and then levels of phosphorylated (p) ERK1/2, total ERK1/2 were determined by Western blotting. β-actin was used as a loading control.
